# Enteral Nutrition during Radiotherapy for Oropharyngeal Cancers: Prevalence and Prognostic Factors Based on HPV Status (A GETTEC Study)

**DOI:** 10.3390/jcm12093169

**Published:** 2023-04-28

**Authors:** Dorian Culié, Renaud Schiappa, Tanguy Pace-Loscos, Bruno Guelfucci, Sebastien Vergez, Renaud Garrel, Nicolas Fakhry, Olivier Dassonville, Gilles Poissonnet, Benjamin Lallemant, Anne Sudaka, Esma Saada-Bouzid, Karen Benezery, Stephane Temam, Phillipe Gorphe, Emmanuel Chamorey, Alexandre Bozec

**Affiliations:** 1 Antoine Lacassagne Centre, University Institute of the Face and Neck, Côte d’Azur University, 06000 Nice, France; 2 Antoine Lacassagne Centre, Department of Epidemiology, Biostatistics and Health Data, Côte d’Azur University, 06000 Nice, France; 3 Department of Otorhinolaryngology and Head and Neck Surgery, Sainte Musse Hospital, 83100 Toulon, France; 4 Department of Otorhinolaryngology and Head and Neck Surgery, Cancer University Institute of Toulouse, 31100 Toulouse, France; 5 Department of Otorhinolaryngology and Head and Neck Surgery, University Hospital of Montpellier, 34295 Montpellier, France; 6 Department of Otorhinolaryngology and Head and Neck Surgery, Public Assistance—Hospitals of Marseille, 13005 Marseille, France; 7 Department of Otorhinolaryngology and Head and Neck Surgery, University Hospital of Nîmes, 30900 Nîmes, France; 8 Antoine Lacassagne Centre, Department of Pathology, Côte d’Azur University, 06000 Nice, France; 9 Antoine Lacassagne Centre, Department of Medical Oncology, Côte d’Azur University, 06000 Nice, France; 10 Antoine Lacassagne Centre, Department of Radiotherapy, Côte d’Azur University, 06000 Nice, France; 11 Department of Otorhinolaryngology and Head and Neck Surgery, Gustave Roussy Institute, 94805 Villejuif, France

**Keywords:** oropharynx, cancer, human papilloma virus, nutrition, feeding tube

## Abstract

Nutritional support during radiotherapy is crucial to tolerating and completing oropharyngeal squamous cell carcinoma (OPSCC) treatment. The impact of HPV status on nutritional support is debated. The objective was to evaluate the rate of Reactive Feeding Tube (RFT) use and determine its prognostic factors during definitive radiotherapy for OPSCC. All OPSCC patients treated from 2009 to 2014 were included in this multicentric retrospective study. The impact of tumor p16 status on the risk of RFT was assessed through multivariate analyses. Among the 543 patients, 103 patients required an RFT (19.0%). The use of RFT differed between centers (5% to 32.4%). In multivariate analysis, only tongue base involvement and concurrent chemotherapy were significantly associated with RFT (OR = 2.18 and 3.7, respectively). Tongue base involvement and concomitant chemotherapy were prognostic factors for RFT. HPV status was not a prognostic factor for enteral nutrition during radiotherapy for OPSCC.

## 1. Introduction

Cancer patients have a major risk of malnutrition, with an estimated prevalence of 50% at diagnosis [1]. Patients with head and neck cancer are particularly at risk, with a primary tumor often responsible for odynophagia and dysphagia [2,3]. Furthermore, treatments, such as radiotherapy, can also increase malnutrition, increasing pain and swallowing difficulties with radiodermatitis and/or radiomucitis [4]. These dietary difficulties can thus lead to weight loss and deterioration of the general condition, leading to treatment interruptions or premature complete cessation, a detrimental element in terms of overall survival [5]. The nutritional care of these patients is, therefore, a major element of the initial overall care.

Among head and neck cancers, HPV oropharyngeal squamous cell carcinoma (OPSCC) is a distinct entity and is also the subject of many clinical trials [6]. HPV OPSCC presents biological, epidemiological, and clinical differences [7]. The incidence of HPV-OPSCC has been increasing in recent decades; it is, in fact, the number one HPV-related cancer in the United States (ahead of cervical and anal cancer) and will be the number one head and neck cancer in the coming decades [8].

Radiotherapy is a significant treatment in the management of OPSCC [9], and nutritional support for these patients is crucial. Similar to other head and neck cancers patients, HPV OPSCC patients are at nutritional risk during radiotherapy [4]. However, few studies have reported on the prognostic factors for the need for a feeding tube during radiotherapy in this specific population. Some authors have suggested that HPV OPSCC patients were more likely to require a feeding tube, while others have not observed any impact [10,11,12].

The objective of this study was to evaluate the rate of Reactive Feeding Tube (RFT) use and to determine its prognostic factors during definitive radiotherapy for OPSCC treatment. 

## 2. Materials and Methods

This retrospective multicenter study involving 7 French tertiary cancer care centers (Nice, Nimes, Villejuif, Marseille, Montpellier, Toulon, and Toulouse) was conducted by the GETTEC (Groupe d’Etude des Tumeurs de la Tête et du Cou) collaborative study group. 

### 2.1. Patients

The inclusion criteria were as follows:

- Previously untreated OPSCC diagnosed between 2009 and 2014;
- OPSCC p16 status determined through immunohistochemistry;
- Curative intent treatment;
- Treatment based on definitive radiotherapy.
- The exclusion criteria were as follows:
- Prior surgical treatment of the tumor or lymph nodes;
- Metastatic OPSCC;
- Undetermined p16 tumor status;
- Medical history of head and neck cancer;
- Medical history of head and neck radiotherapy;
- Feeding tube before treatment initiation.

### 2.2. Ethical Considerations

The study was conducted in accordance with the Declaration of Helsinki. In accordance with French legislation, insofar as this study complies with the MR004 legislation on retrospective studies, the patients were informed of the conduct of this study. The study was declared to the French Health Data Hub, and the database was declared to the French National Commission for Information Technology and Liberties (database declaration number: 199655v0).

### 2.3. Study Measurements 

Patients’ comorbidity levels were determined using the American Society of Anesthesiologists (ASA) and the Kaplan–Feinstein index (KFI). Tobacco and alcohol consumption were noted. Patients were staged according to the 2009 (7th edition) American Joint Committee on Cancer staging system. The data recorded included the anatomical subsite of the index OPSCC (base of tongue, lateral pharyngeal wall, glossotonsillar sulcus, posterior pharyngeal wall, and soft palate) and the insertion and type of enteral feeding tube during radiotherapy (nasogastric tube and percutaneous gastrostomy tube).

The immunodetection of the protein p16 was used as a surrogate marker of HPV tumor status. Routine formalin-fixed, paraffin-embedded, pretreatment tumor tissues were tested for p16 expression through the use of immunohistochemistry. As usually recommended [13], tumors were classified as p16+ in the case of strong, diffuse nuclear and cytoplasmic staining in more than 70% of carcinoma cells, while tumors displaying unconventional keratinizing morphology, together with a variable or patchy p16 staining pattern, were classified as p16−. 

The patients were classified into two groups according to nutritional management:

- The absence of enteral nutrition during or after treatment;
- The implementation of a Reactive Feeding Tube (RFT) during or after treatment.

The implementation of an RFT was decided during a consultation with a member of the healthcare team (head and neck surgeon, general practitioner, radiotherapist, or nutritionist) based on weight, body mass index (BMI), and food intake. Thus, if the weight loss exceeds 5%, the risk of malnutrition is significant and, therefore, requires intervention [14].

### 2.4. Statistical Analyses

The distribution of the patient’s clinical characteristics according to p16 tumor status was analyzed in the univariate analysis. We investigated the impact of p16 tumor status and other relevant clinical factors (gender, age, ASA score, KFI score </≥2, T/A consumption, T-stage, N-stage, and tumor anatomical subsite) on the risk of RFT implementation in univariate analyses using Chi-2 tests, as confirmed by Fisher’s exact tests. For multivariate analyses, all of the variables associated with a *p* ≤ 0.10 in the univariate analysis were included in the logistic regression models. 

Overall survival (OS) was defined from the date of diagnosis to death due to any reason or censored if patients were alive at the last follow-up. OS was determined using Kaplan–Meier analysis. To analyze the impact of an RFT on patient survival, we compared OS in patients with and without RFT using a Log-Rank test. 

All of the statistical analyses were performed at 5% alpha risk or a 95% confidence interval by the biostatistician through the use of R.3.0.1 software on Windows (R Foundation for Statistical Computing, Vienna, Austria).

## 3. Results

### 3.1. Patients Clinical Characteristics

A total of 543 patients, 403 men and 140 women, (mean age 61.6 ± 9.1 years), were included in the present study. The main clinical characteristics for the whole cohort and according to the p16 tumor status are shown in Table 1. The median follow-up was 41.4 months. Forty patients had a KFI score of 3 (12 patients in the p16+ group and 28 in the p16− group), and 21/40 received concurrent chemotherapy.

### 3.2. Risk and Predictive Factors of RFT Implementation According to Initial Features

In the cohort, 103 patients required an RFT (19.0%). The comparison of the baseline clinical characteristics according to the placement of an RFT is reported in Table 2.

In the univariate analysis, we observed differences between the different centers. Indeed, some have made greater use of RFTs (5% to 32.4%). Patients requiring RFTs were older (*p* = 0.076). The tumors of patients requiring RFTs more frequently invaded the glossotonsillar sulcus (*p* = 0.031) and the base of the tongue (*p* = 0.027). The initial N stage of patients requiring an RFT was higher (*p* = 0.014). In the multivariate analysis, only tongue base involvement was significantly associated with the need for an RFT (*p* < 0.01; OR = 2.18; 95% confidence interval (CI) = 1.18–4.08).

### 3.3. Risk and Predictive Factors of RFT Implementation According to Therapeutic Modalities

The therapeutic modalities and major acute toxicities between the two groups are reported in Table 3. Patients receiving chemotherapy concomitantly with radiotherapy had a greater frequency of requiring an RFT (*p* < 0.001; OR = 3.7; 95% confidence interval (CI) = 1.35–12.37). Local toxicity (mucositis *p* = 0.01, dysphagia *p* < 0.001) was significantly associated with RFTs. Patients with RFTs had a greater number of days off-treatment (*p* = 0.011).

### 3.4. Oncological Results

**Figure 1:**
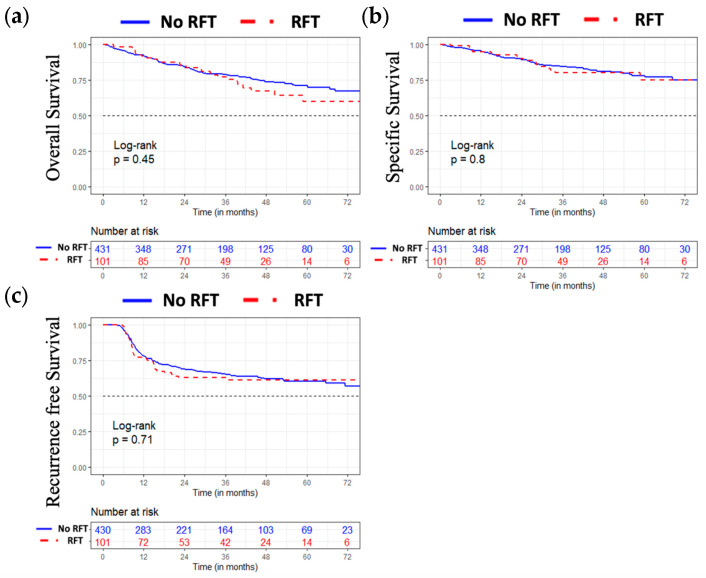
Overall (**a**), specific (**b**), and recurrence-free survival (**c**) according to the implementation of an RFT.

The overall, specific, and recurrence-free survival of the entire population studied according to the nutritional strategy used are illustrated in Figure 1. We did not observe any differences between the two groups in terms of overall, specific, and recurrence-free survival.

### 3.5. Proportion of RFT According to the Number of Risk Factors (Figure 2)

We established a prognosis score, based on previous significant results, to assess the use of RFT. Thus, the rate of RFT use for patients with a score of zero is 4.2% compared to 28.6% for those with a score of 3.

## 4. Discussion

In our study, the main clinical differences observed between HPV and non-HPV OPSCC are in agreement with the results previously published in the literature [8]. 

Several studies have evaluated the nutritional strategies used for patients with head and neck cancer treated with radiotherapy and/or chemotherapy [15,16,17]. In our series, we observed the establishment of RFTs for 19.0% of the patients. This rate of enteral nutrition is consistent with certain series [18,19], lower [20], or, on the contrary, much higher than others [21], and there is, in fact, a large variation in the rate of enteral nutrition during radiotherapy treatment for head and neck cancers, varying from 15% [22] to more than 70% [23]. Most studies include all head and neck cancers, thus encompassing many anatomical locations. The lack of analysis by location limits the comparison of results from one series to another, as each location has a different functional impact on swallowing [24]. This heterogeneity of results also highlights the lack of consensus regarding nutritional management, with heterogeneity of management according to local habits. This heterogeneity underlines the need to identify precise prognostic factors in order to detect patients who will need nutritional support early. 

The involvement of the base of the tongue was one of the prognostic factors for the implementation of enteral nutrition during radiotherapy (chemotherapy). The base of the tongue is composed of numerous muscles whose contraction allows the propulsion of the food bolus in the pharyngeal funnel [25,26,27]. Its contraction is, therefore, mandatory during any swallowing movement, a function rapidly altered in the case of irradiation [28]. Because each site of the aerodigestive tract has its own function, several studies have focused on the locations most at risk for dysphagia during radiation therapy. The oropharynx and hypopharynx have already been described as two sites at risk for leading to malnutrition in patients with head and neck cancer [2]. However, to our knowledge, no study has specifically studied the functional units of the oropharynx. The pejorative character of the tongue base has, therefore, never been described.

In our series, the mean age of patients who required an RFT was greater than that of patients who did not, although this difference was not significant. It is recognized in the literature that elderly patients represent a population at risk for malnutrition [29], especially in the case of head and neck cancers [30]. Similarly, we find in our study that the initial N stage of patients requiring an RFT was more advanced (*p* = 0.014) in the univariate analysis. Although this significance was not confirmed through the use of multivariate analysis, it would appear that patients with significant lymph node involvement are at greater risk of malnutrition. Other authors have also demonstrated the negative impact of the advanced lymph node stage on feeding ability during radiotherapy [31]. Indeed, these patients are subjected to higher doses and volumes of irradiation at the cervical level, which leads to increased local toxicity. Anderson et al. [32] correlated the rate of enteral feeding with the doses/volumes received. Although the emergence of intensity modulation for head and neck cancer irradiation in recent years has significantly reduced acute and late toxicities [33], nodal irradiation irradiates the underlying pharyngolarynx [34] due to its anatomical proximity. It is also a major functional unit in swallowing, whose irradiation can lead to dysphagia [33]. Finally, lymph node involvement usually leads to an advanced-stage classification of the tumor according to the 2009 American Joint Committee on Cancer TNM classification. The advanced stage usually requires the addition of chemotherapy to radiation therapy. Therefore, node invasion may be associated with a higher rate of enteral nutrition due to the requirement of chemotherapy to potentiate radiotherapy.

In our series, we observed that patients requiring an RFT more frequently received concomitant chemotherapy. Concomitant chemotherapy is traditionally indicated in cases of locally advanced stage (T3 or T4) and/or lymph node involvement (N+) [9] in addition to radiotherapy. The desired effect of chemotherapy is to potentiate the local action of radiotherapy in order to improve the oncological results [19,35,36,37]. Thus, by potentiating radiotherapy, chemotherapy also potentiates acute and late side effects [38,39]. In an extensive literature review published in 2015, Bossola emphasized the importance of nutritional support during chemoradiotherapy for the treatment of head and neck cancer [40]. Several studies have reported a greater use of enteral nutritional support for patients receiving chemoradiotherapy compared to radiotherapy alone [41,42]. In addition, in a recent U.S. retrospective study of 192 head and neck cancer patients treated with (chemo)radiation therapy between 2010 and 2012, the authors demonstrated that chemotherapy was significantly associated with the placement of a percutaneous gastrostomy [43]. 

Few studies have specifically addressed the impact of HPV status on the use of enteral nutrition during radiation therapy for oropharyngeal cancer. The main published series is that of Vangelov et al. [44]. In this single-center Australian retrospective study, the authors included 100 patients treated with radiation therapy for oropharyngeal cancer between 2011 and 2016, including patients receiving adjuvant radiation therapy after surgical treatment. Sixty-eight patients had a positive HPV status, 10 were negative, and 22 were unknown. Sixty-one patients required enteral nutrition, RFTs in the majority of cases (33/61, 54%). The authors reported greater feeding tube use for patients with HPV-induced OPSCC (43/68, 63%) than for other patients (18/32, 56%). This difference was not significant in the multivariate analysis. Despite a small number of HPV-negative patients, the authors established a probability model to conclude that the nature of HPV-induced cancers, as well as concomitant chemotherapy, were factors associated with the need for a feeding tube. In a recent review of the literature, Brewczynski et al. [10] reported all publications evaluating nutritional modalities during radiation therapy for OPSCC according to HPV status. They described some studies that observed greater weight loss and more frequent use of enteral nutrition in patients with HPV-induced OPSCC. These series were all retrospective, with these observations sometimes made within subgroups. Moreover, as in the study by Vangelov et al. [11], these studies primarily involved cohorts composed of >90% of HPV-induced OPSCCs, which limits any comparability. No other clinical prognostic criteria for RFTs were described in these two studies. The main explanation provided by the different authors was based on a probably higher acute local toxicity in patients with HPV-induced OPSCC compared to patients with HPV-negative OPSCC [44]. However, this notion is also debated in the literature as other authors have demonstrated similar toxicity regardless of HPV status [12]. From our perspective, there is no justification for the increased use of RFTs based on HPV status. As discussed above, tongue base involvement is one of the major prognostic factors. Thus, the preferential involvement of the lingual and palatine tonsils in HPV OPSCC may explain why this population is more at risk from a nutritional point of view [45]. Harrowfield et al. [46] recently published a series from a prospective observational study subgroup. In this small heterogeneous series (70 patients with HPV-induced OPSCC versus 13 with HPV-negative OPSCC, including patients with surgery plus adjuvant radiotherapy and patients with prophylactic enteral tube), the authors observed no difference in weight loss or more frequent use of RFTs according to HPV status.

In our series, we observed that patients who required RFTs had greater treatment discontinuation than those who did not, although this difference was not significant in the multivariate analysis. This is one of the reasons why some authors recommend the use of a prophylactic feeding tube. Indeed, the prophylactic placement of a feeding tube makes it possible to avoid any therapeutic pause during radiotherapy [47,48,49]. It has been described that treatment interruption during radiotherapy for head and neck cancer is a factor in poor prognosis [50]. In our series, however, this difference was small (1 day), emphasizing the need for close monitoring and responsiveness in nutritional management [40]. Therefore, we assessed the potential impact on survival, and we did not observe any differences in survival between the two groups. 

The decision to use a prophylactic or reactive feeding tube (RFT) is a never-ending debate [13,51,52,53,54]. Our study does not provide evidence of a comparison between these two strategies. 

We report here the largest series focusing on the nutritional modalities of patients during radiotherapy treatment for OPSCC. Its multicenter nature is also an asset as it limits the center effect of each hospital, even if it may also represent a bias since the decision to use an RFT was always made by each care team. The large number of centers included (seven), and the non-significant aspects in the multivariate analysis of this variable, limit this bias. The retrospective nature, limiting access to accurate data on comorbidities, and the lack of additional information on nutritional status (weight curves, body mass index, the quantity of food ingested orally, blood nutritional assessment, etc.) constitute the limitations of this study. The lack of accurate information concerning dosage, rate of administration, and type of platinum salts (carboplatin or cisplatin) may also be a bias and should be specifically investigated in the future. In addition, the radiation technique was not reported, although all centers were equipped with intensity-modulated radiation therapy during this period. In addition, although a higher rate of RFT use was observed in 2014, the year of treatment was not a prognostic factor for RFT use in the multivariate analysis. However, no prospective studies have been published on the subject, and additional information on the nutritional status of patients is frequently missing from electronic medical records.

## 5. Conclusions

In our study, tongue base involvement, age, and concomitant chemotherapy were prognostic factors for the need for an RFT. In contrast, we observed that HPV status does not appear to be a prognostic factor for enteral nutrition during radiotherapy for oropharyngeal cancers. In all cases, these patients require close follow-up in an adapted care structure, in close collaboration with all members of the medical team. Overall, it appears that further adequate prospective, randomized studies are needed to validate these results.

## Figures and Tables

**Figure 2:**
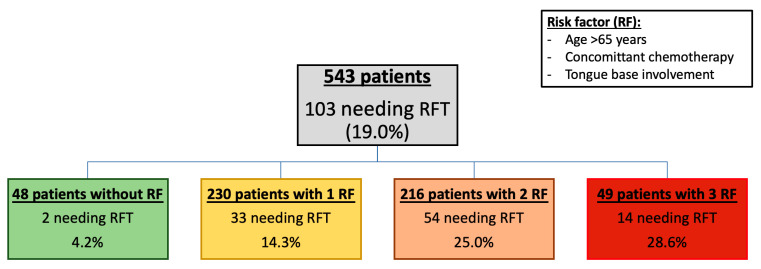
Rate of patients with an RFT according to the risk factors identified.

**Table 1:** Description of initial clinical characteristics and treatment modalities among p16+ and p16− patients.

	All Patients N = 543 (%)	p16+ Patients N = 250 (%)	p16− Patients N = 293 (%)	*p* Values (UA)
***Initial clinical features***				
**Gender:** male/female	403 (74.2)/140 (25.8)	181 (72.4)/69 (27.6)	222 (75.8)/71 (24.2)	NS
**Age:** average ± SD	61.08 ± 9.91	61.87 ± 9.88	60.45 ± 9.86	NS
**Age:** >65/≥65 years	369 (68)/174 (32)	156 (62)/94 (38)	213 (73)/80 (27)	0.01
**ASA score:** </= 3	392 (77.8)/112 (22.2)	200 (80.0)/36 (14.4)	192 (65.6)/76 (25.9)	NS
**KFI score:**	153C(28.4)/270 (50)/	105 (42.2)/104 (42)/	48 (16.7)/166 (57)/	NS
0/1/2/3	80 (15)/40 (7.4)	29 (12)/12 (4.8)	51 (17)/28 (9.6)	
**Tobacco use:** </≥10 PY	189 (38.3)/304 (61.7)	104 (41.6)/146 (58.4)	85 (29.0)/208 (71.0)	0.03
**Tumor site:** BOT/LPW/GTS/PPW/SP	199 (36.7)/346 (63.7)/156 (28.7)/35 (6.5)/139 (25.6)	95 (38.0)/188 (75.2)/71 (28.4)/3 (1.2)/47 (18.8)	104 (35.5)/158 (53.9)/85 (29.0)/32 (10.9)/92 (31.4)	NS/0.02/NS/<0.001/<0.001
**T stage:** </≥ 3	225 (41.5)/317 (58.5)	123 (49.2)/127 (50.8)	102 (34.8)/190 (64.8)	NS
**N stage:** </≥ 2a	207 (38.6)/330 (61.5)	79 (31.6)/169 (67.6)	128 (43.7)/161 (54.9)	0.06
***Treatment modalities***				
**Year of treatment:**				NS
2009	43 (7.9)	19 (7.6)	24 (8.2)	
2010	61 (11.2)	17 (6.8)	44 (15.0)	
2011	93 (17.1)	43 (17.2)	50 (17.1)	
2012	106 (19.5)	47 (18.8)	59 (20.1)	
2013	107 (19.7)	52 (20.8)	55 (18.8)	
2014	122 (22.5)	67 (26.8)	55 (18.8)	
**Induction CT:** yes/no	116 (21)/427 (79)	44 (18)/206 (82)	72 (25)/221 (75)	0.048
**Conco-CT:** yes/no	435 (80.1)/108 (19.1)	210 (84.0)/40 (16.0)	225 (76.8)/68 (23.2)	0.04
**RT interruption:** Mean days ± SD	0.78 ± 3.8	0.74 ± 3.8	0.73 ± 3.8	NS
Mucositis toxicity *: <2/≥2	322 (59.3)/221 (40.7)	135 (54.0)/115 (46.0)	187 (63.8)/106 (36.2)	0.02
Dysphagia toxicity *: <2/≥2	412 (75.9)/131 (24.1)	179 (71.6)/71 (28.4)	233 (79.5)/60 (20.5)	0.03
Dyspnea toxicity *: <2/≥2	540 (99.4)/3 (0.6)	249 (99.6)/1 (0.4)	291 (99.3)/2 (0.7)	NS

SD: standard deviation; NS: not significant; ASA: American Society of Anesthesiologists; KFI: Kaplan Feinstein Index; PY: pack years; Tumor site: BOT: base of tongue; LPW: lateral pharyngeal wall; GTS: glossotonsillar sulcus; PPW: posterior pharyngeal wall; SP: soft palate, Conco-CT: concomitant chemotherapy, RT: radiotherapy, *: CTCAE scale; UA: comparison between p16+ and p16− patients in univariate analysis.

**Table 2:** Initial clinical characteristics of patients with and without an RFT.

Clinical Features	No RFT N = 440 (%)	RFT N = 103 (%)	*p* Values (UA)	*p* Values (MA)
**Center:** Nice/Nîmes/ Villejuif/Marseille/ Montpellier/Toulon/Toulouse	80 (70.8)/57 (89.1)/100 (67.6) /37 (94.9)/40 (90.9)/ 38 (95)/88 (92.6)	33 (29.2)/7 (10.9)/48 (32.4)/2 (5.1)/4 (9.1)/ 2 (5)/7 (7.4)	<0.001	NS
**Gender:** male/female	326 (80.9)/114 (81.4)	77 (19.1)/26 (18.6)	0.989	-
**Age:** average ± SD	60.71 ± 9.83	62.65 ± 10.15	0.076	NS
**Age:** >65/≥65 years	305 (69)/135 (31)	64 (62)/39 (38)	0.2	-
**ASA score:** </=3	311 (79.3)/92 (82.1)	81 (20.7)/20 (17.9)	0.603	-
**KFI score:** /0/1/2/3	124 (27.7)/217 (49)/ 62 (14)/36 (8.4)	29 (28)/53 (51)/ 18 (17)/3 (2.9)	0.675	-
**Tobacco use:** </≥10 PY	154 (81.5)/241 (81.4)	50 (18.6)/52 (19.4)	0.892	-
**Tumor site:** BOT/LPW/GTS/PPW/SP	151 (75.9)/278 (80.3)/117 (75)/28 (80)/116 (83.5)	48 (24.1)/68 (19.7)/39 (25)/7 (20) /23 (16.5)	0.027/0.671/0.031/1/0.472	0.01/-/NS/-/-
**T stage:** </≥3	187 (83.1)/252 (79.5)	38 (16.9)/65 (20.5)	0.344	-
**N stage:** </≥2a	179 (86.5)/256 (77.6)	28 (13.5)/74 (22.4)	0.014	NS
**p16 status:** positive/negative	196 (78.4)/244 (83.3)	54 (21.6)/49 (16.7)	0.182	NS

SD: standard deviation; NS: not significant; ASA: American Society of Anesthesiologists; KFI: Kaplan Feinstein Index; PY: pack years; Tumor site: BOT: base of tongue; LPW: lateral pharyngeal wall; GTS: glossotonsillar sulcus; PPW: posterior pharyngeal wall; SP: soft palate, CT: chemotherapy, RT: radiotherapy; UA: comparison between patients with and without RFT in univariate analysis, MA: comparison between patients with and without RFT in multivariate analysis.

**Table 3:** Treatment modalities of patients with and without an RFT.

Therapeutic Modalities	No RFT N = 440 (%)	RFT N = 103 (%)	*p* Values (UA)	*p* Values (MA)
**Year of treatment:**			<0.001	NS
2009	40 (9.1)	4 (3.9)		
2010	57 (13)	5 (4.8)		
2011	79 (18)	17 (16.3)		
2012	92 (20.9)	16 (15.4)		
2013	89 (20.2)	20 (19.2)		
2014	82 (18.6)	41 (39.4)		
**Induction CT:** Yes/No	95 (22)/345 (78)	21 (20)/82 (80)	NS	-
**Concomitant CT:** Yes/No	340 (78.2)/100 (92.6)	95 (21.8)/8 (7.4)	0.001	0.01
**Concomitant CT:**				-
Platinum salts	200 (75.2)	66 (24.8)	0.626	
Cetuximab	81 (81)	19 (19)	0.231	
Other	15 (65.2)	8 (34.8)	0.313	
**RT interruption:** Mean days ± SD	0.54 ± 3.2	1.7 ± 6.07	0.01	-
Mucositis toxicity *: <2/≥2	72 (80)/142 (64.3)	18 (20)/79 (35.7)	0.01	-
Dysphagia toxicity *: <2/≥2	148 (82.2)/66 (50.4)	32 (17.8)/65 (49.6)	<0.001	<0.0001
Dyspnea toxicity *: <2/≥2	212 (69.3)/2 (66.7)	94 (30.7)/1 (33.3)	1	-

SD: standard deviation; NS: not significant; CT: chemotherapy, RT: radiotherapy, *: CTCAE scale; UA: comparison between patients with and without RFT in univariate analysis; MA: comparison between patients with and without RFT in multivariate analysis.

## Data Availability

The data used for this study is not made available to the public.
